# Pharmacopœia of the American Institute of Homœopathy

**Published:** 1897-08

**Authors:** 


### BOOK NOTICES.

PHARMACOPŒIA OF THE AMERICAN INSTITUTE OF HOMŒOPATHY. Published for the Committee on Pharmacopœia of the American Institute of Homœopathy. Boston: Otis Clapp & Son, Agents, No. 10 Park Square. 1897. 1 vol., 676 pages. Price, cloth, $4.25 net; half morocco, $5 net. Delivered to any part of the United States at above prices.

The homœopathic school has long been in need of an authoritative pharmacopœia; one that would be accepted by all pharmacists and all physicians in the profession.

Boericke & Tafel published such a work, edited by Dr. Joseph T. O'Connor. But, unfortunately, though a most admirable book, and accepted by a large number in the profession, as is shown by its passing through a third edition, yet it is not considered as binding upon the whole school. Hence a variety of methods of preparing drugs has sprung up with some differences of notation.

The American Institute of Homœopathy, keenly realizing this confusing state of affairs, and desiring to reduce it to order and bring the whole subject up to a scientific standard, appointed a committee of twelve members, of whom six were pharmacists, to consider the question and devise a plan which would enable them to publish a book which should be considered the authority of the homœopathic school.

The Institute, being one of the largest bodies of medical men in the world, and having among its members every shade of variation in opinion upon the truth of Homœopathy, carries with it a moral force which must cause all to feel its deliverances binding upon them, and thus slowly and surely it must bring about the much-desired uniformity.

One of the most striking characteristics of the book, and one which will at once arrest attention, is the endeavor to establish a "unit of medicinal strength." With this object in view "the committee have in all cases made the dry crude drug the unit from which to estimate strength. The plant moisture is to be regarded as a part of the vehicle or menstruum, it being evident that the water contained in the plant is but a solvent, and forms no part of its medicinal substance."

The mother tincture, on this plan, is considered to represent the first decimal dilution, and is thus brought into corresponding strength with the first trituration. "Uniformity is thus secured, and the signs 1x or $\frac{1}{10}$. on whatever form of attenuation they may be found, will always represent a drug power of one-tenth. The 2x will show the presence of $\frac{1}{100}$ part drug substance, and the familiar 3x will show $\frac{1}{1000}$ part."

The amount of plant moisture naturally existing in the plant must be found by a careful assay of a portion of the plant for water, and if it be less than normal, water is to be added to raise it to the standard, and then the preparation thus obtained is to be called the first decimal dilution.

The reader is not to infer from the foregoing that the plant is first to be dried and then to be put into the tincture-making menstruum. On the contrary, the committee distinctly say, and more than once repeat, that "it should be understood, however, that the fresh green materials are still required in the preparation of tinctures."

This scheme for the determination of the value of dilutions approaches the ideal of scientific accuracy. It must, however, cause some confusion in the minds of those who already have for sale, or for use in their practice, dilutions made in other ways. This confusion, however, is of but little moment, in view of the diversity of practice in making these preparations already practiced by different pharmacists, and which already amounts to confusion. Even under these circumstances the difference between the notation of the Institute as proposed and that actually practiced by most pharmacists amounts to only one dilution.

The confusion will, in time, disappear as a standard is now set up by which all who assume to prepare medicines for homœopathic practice may be guided. To those who use the thirtieth and higher dilutions or potencies this question will have but little weight, as in any case it can have but inconceivably small influence in the value of the fractions representing such potencies.

The writer of this review may be pardoned for expressing a doubt as to whether this ideal uniformity has been attained by the plan of the committee. It would require a most elaborate and exhaustive investigation by means of chemical analysis to establish beyond dispute the certainty of the object being attained. But even if it be doubtful the scheme is a long advance toward accuracy, and should be strongly sustained rather than opposed, as seems likely at present.

Turning now to the general plan of the book, we find it divided into three general parts. The first part treats of general pharmacy and of the above-mentioned scheme of making attenuations. The second part treats of special pharmaceutics; and the third gives various tables of weights and measures and equivalent values of weights and measures in English and French systems. Then there is a list of medicines with correct pronunciation indicated, and page where they may be found; and finally there is a most complete index of fifty-two double-column pages.

In the second part, "Special Pharmaceutics," the medicines are given in alphabetical order, so that it is possible to find any one wanted instantly without recourse to any index.

Medicines like Psorinum, Medorrhinum, and Tuberculinum are not admitted. In fact every one of the nosodes is ignored. We predict that this omission will, in time, be remedied.

All medicines admitted to the list are carefully and thoroughly described, so that there is no mistaking them.

Actea-racemosa appears under its better known name Cimicifuga, and Calcarea as Calcarea-carbonica instead of Calcarea-ostrearum, as it is in Hering's *Guiding Symptoms.*

Taking it altogether, the *Pharmacopœia* must hold in the homœopathic profession the same place that is held in the old school of medicine by that noble magazine of learning, *The United States Dispensatory*.

